# Machine Learning Assisted Hybrid Cuckoo Search for Predictive Optimization in Renewable Energy Systems

**DOI:** 10.12688/f1000research.172760.2

**Published:** 2026-04-24

**Authors:** Noor Dalaf, Iraq T. Abbas

**Affiliations:** 1 Mathematics, University of Baghdad Al-Jaderyia Campus College of Science, Aljaderia, -- US or Australian state or Canadian province --, 00964, Iraq

**Keywords:** Hybrid Cuckoo Search, Machine Learning, Predictive Optimization, Renewable Energy Systems, Metaheuristic Algorithms, Smart Energy Forecasting, Energy Management, Computational Intelligence, Optimization Techniques, Intelligent Decision Support

## Abstract

**Background:**

Due to the intermittent, nonlinear, and uncertain behavior of renewable energy sources (res) such as solar and wind, grid stability and reliability require very high forecasting and optimization skills as widely reported in the literature. Traditional optimization methods work very well in small or static systems but are suffer difficulty on large-scale, dynamic and stochastic renewable environment due to their NP-hard nature.

**Methods:**

The framework introduces the concept of a Machine Learning-Assisted Hybrid Cuckoo Search (ML-HCS) that combines CS with a hybrid metaheuristic and integrates Long Short-Term Memory (LSTM) networks for forecasting based on both regression models of LSTMs and hybrid optimization algorithms. LSTM model produces predictive signals that help inform the search trajectory of CS, enabling better exploration–exploitation tradeoff of resource scheduling on uncertainty.

**Results:**

Simulation experiments on benchmark renewable energy datasets showed that ML-HCS not only converges 12% faster than the best of the GA, PSO, and classical CS, but also achieves 7–10% better quality of solutions and 9% higher robustness. This model also adapted better in multi-objective optimization tasks: cost minimization, scheduling stability and prediction accuracy.

**Conclusions:**

Finally, the ML-HCS framework provides a prediction-oriented, data-driven, scalable optimization methodology for renewable energy systems. Its use of machine learning and metaheuristic search provide for high forecasting accuracy and resiliency in operation, which will enable its future large scale smart grid and renewable energy management applications.

## 1. Introduction

The rapid penetration of RES into contemporary power grids demands smart forecasting and optimization techniques to avoid stability, reliability, and cost problems in the grid. With sustainability and plenty in their favor, especially solar and wind (photovoltaic (PV)), they are the most promising contenders; however, the stochastic and intermittent nature of these energies is a big challenge for reliable supply-demand balancing. Various optimization techniques such as linear programming, Genetic Algorithms (GA), and Particle Swarm Optimization (PSO), have been traditionally employed for scheduling and resource allocation. These methods are ideal for small or static systems but prove inappropriate when dealing with systems that are large, complex, dynamic, and uncertain in nature owing to the nonlinearity and multimodality of the solution space. ^1, 2^

To address this type of design problem, metaheuristic algorithms have gained considerable power in the last few years. Thus far, Cuckoo Search (CS) has attracted a wealth of attention because it has a strong global search capability and is a simple algorithm with relatively few parameters. ^3^ These features can always be advantageous, but the conventional CS is still hampered by the parameter settings forming premature convergence and sensitivity to parameter tuning, which usually induce complications in practice in renewable energy optimization problems. ^4^ Specifically, on the one hand, Machine Learning (ML) has a remarkable performance in forecasting the generation of renewable energy and the demand for load, where ML, such as time series forecasting models based on deep learning, such as Long Short-Term Memory (LSTM) and Gated Recurrent Units (GRU), are used. The ability of the models to capture nonlinear dynamical non-stationarities can render them particularly suitable for intermittent and non-predictable renewables. ^5, 6^

Thus, it allows for a strong hybrid architecture of predictions, along with metaheuristic optimization. In this frame, ML makes precise anticipation for request and creation and methodology such as CS enhances scheduling and allotment. Therefore, this reduces the computational cost exponentially and increases the speed of convergence, as it helps direct the search towards the most promising areas of the solution space. ^7, 8^ In this regard, this work proposes a new ML-HCS, which is a recent ML–HCS algorithm for the predictive optimization of RES. Owing to such limitations of the traditional approaches, we developed a framework that is motivated by the need to utilize the advantage of ML for prediction and CS for exploration exploitation trade-offs. ^9, 10^ It will prove essential to boost the stability of the grid, reduce the cost of operation, and scale them for smart grid applications in the future.

## 2. Literature review

The motivation for writing this paper comes from the growing body of research on renewable energy forecasting and optimization. ^3, 11– 14, 16^ In recent years, smart energy management in large-scale power systems has gained substantial interest ^17, 18^; thus, accurate forecasts of renewable energy have recently gained research attention. ^19^ Various machine learning methods, ^5, 20– 22^ including Artificial Neural Networks (ANN), Support Vector Regression (SVR), ^23^ and Recurrent Neural Networks (RNN), ^9^ are widely used for short- and long-term forecasting of renewable energy generation. Such models can reproduce nonlinear dynamics and their time-varying nature; both are important features that need to be captured when accounting for the stochastic nature of solar and wind resources.

On the other hand, metaheuristic algorithms such as Genetic Algorithms (GA), ^24^ Particle Swarm Optimization (PSO) ^25^ and Cuckoo Search (CS), ^26^ are focused on solution discovery for large-scale scheduling and optimization issues in energy systems. Although these approaches offer more flexible options, in practice they are usually subject to shortcomings such as premature convergence or sensitivity towards parameter tuning hence decreasing robustness under high uncertainty environments. ^27, 28^

More recently, research has focused on hybrid frameworks that combine forecasting techniques with optimization algorithms. PSO–ANN and GA–ANN systems, for example, exhibit synergistic effects that can significantly enhance scheduling efficiency and cost reduction over standalone techniques [32]. Indeed, the proposed combination of machine learning forecasting models under Cuckoo Search is still very novel. Table 1 Comparison of classical mathematics and ML-HCS in terms of convergence speed, solution quality, and robustness.

**Table 1.** Comparative performance of optimization algorithms.

Algorithm	Convergence speed (%)	Solution quality (%)	Robustness (%)
GA	75	70	68
PSO	80	78	75
CS	85	82	80
ML-HCS	95	93	92

In this comparison we presented in the following table ( Table 2), we compared the results of the ML-HCS algorithm to the (GA), (CS), (PSO), and (Hybrid GA–CS) classical metaheuristics on optimal and average performance indicators, including the makespan *C* max , total tardiness $\Sigma T_{i}$ , maximum lateness Lmax , Accuracy and robustness factors.

Overall, ML-HCS scored the lowest max , Lmax and tardiness and exhibited the highest and lowest accuracy and standard deviation, respectively, demonstrating both efficiency and stability.

The table summarizes the convergence speed, solution quality, and robustness of the evaluated algorithms. The proposed ML-HCS method achieved the highest overall performance, demonstrating faster convergence and robust solutions compared to traditional GA, PSO, and CS approaches.

More detailed comparison: While Table 1 provides a more general overview of comparative metrics between algorithms, Table 2 further evaluates the performance of ML-HCS in combination with task-specific performance measures such as makespan, tardiness, and robustness, yielding further insight into the strengths of the ML-HCS approach in comparison with other hybrid methodologies.

**Table 2.** Comparative performance of ML-HCS with baseline methods.

Algorithm	Cmax (Makespan)	$\Sigma T_{i}$ (Total Tardiness)	Lmax (Max Lateness)	Accuracy (%)	Robustness (Std. Dev.)
GA	125	360	95	86.5	±6.4
CS	118	340	90	88.2	±5.9
PSO	115	330	88	89.0	±5.2
Hybrid GA–CS	108	300	80	91.4	±4.7
ML-HCS	98	270	70	95.8	±3.1

Our results from the simulation experiments on benchmark solar and wind datasets are presented in Tables 1 and 2, respectively, instead of the numbers taken from past studies. To mitigate confusion, this clarification has now been included in the captions.

Conclusion: Machine learning and metaheuristic algorithms individually improve the forecasting and optimization of renewable energy systems, but the research gap needs to be filled to integrate predictive power with optimization. To date, most hybrid frameworks are capable of solving one or two streams of complementary objectives, and only a few have been designed to deal explicitly with the intermittent and uncertain nature of renewables. This leaves significant gaps, and the ML-HCS framework aims to provide an integrated approach that caters to both forecasting accuracy and optimization robustness, specifically for renewable energy applications.

From the above optimization algorithm comparisons, which is shown in Table 1, we can observe that the proposed HMPCS–ML framework yields higher accuracy and convergence in the above table comparing to methods adapt from traditional approaches.

Table 3 presents the Wilcoxon signed-rank test results for pairwise comparisons between ML-HCS and the baseline algorithms. The reported p-values confirm that the proposed method consistently outperforms the competing approaches under the tested conditions.

**Table 3.** Wilcoxon test results between ML-HCS and baseline methods.

Comparison	p-value	Result
ML-HCS vs GA	0.012	Significant
ML-HCS vs PSO	0.018	Significant
ML-HCS vs CS	0.025	Significant

As shown in Table 3, the Wilcoxon test results suggest that the proposed ML-HCS framework provides consistent performance improvements compared to the baseline methods, although the statistical significance may vary depending on the dataset.

**Scientific Interpretation:** Convergence Speed (%): GA recorded 75%, reflecting slower convergence due to limited exploration. PSO improved to 80% through a swarm-based search. The CS reaches 85% with Lévy flights, while the ML-HCS achieves the highest speed at 95%, demonstrating superior global exploration guided by ML predictions.

Solution Quality (%): GA lags at 70% and is often trapped in local optima. PSO and CS improved to 78% and 82%, respectively. ML-HCS outperformed all the methods by 93%, producing higher-quality solutions under uncertainty.

Robustness (%): GA scores were the lowest at 68%, showing unstable outcomes. The PSO and CS were moderately stable at 75% and 80%, respectively. The ML-HCS demonstrated the highest robustness (92%), confirming its stability and consistency. The results confirm that the ML-HCS surpasses the GA, PSO, and CS across all three key metrics: convergence speed, solution quality, and robustness. The integration of machine learning prediction with CS exploration ensures faster convergence, superior solution quality, and enhanced stability, making it highly suitable for large-scale renewable energy optimization problems.

Table 2 Comparison of GA, PSO, CS, and the proposed ML-HCS in convergence speed, solution quality and robustness. These values are derived from simulation experiments on the benchmark solar, wind datasets to make fair comparisons across all methods rather than using different outputs from the literature.

Note: The results demonstrate that ML-HCS achieves the best performance across all metrics, significantly reducing makespan, total tardiness, and maximum lateness, while also achieving higher accuracy and robustness compared to the baseline methods.

## 3. Methodology

Our proposed ML-HCS framework combines forecasting with optimization via a two-stage design with the aim of simultaneously improving the predictive performance and optimization robustness.

### (1) Data sources

The data used in this study were obtained from publicly available solar irradiation and wind power benchmark datasets (i.e., NREL, 2020, Global Energy Forecasting Competition datasets). ^24, 25^ Relevant datasets contain hourly time-series data of generation and load demand for several years with a realistic representation of renewable energies. ^26^ During data preprocessing, normalization (min–max scaling) was used for numerical stability, and missing values were interpolated using linear interpolation techniques. ^27^

### (2) Machine Learning forecasting (LSTM)

The first step involves training LSTM neural networks to forecast the short-term future load demand and renewable generation profiles. The LSTM model architecture includes the following.

These input layers are related to historical time-series data (generation, load, and weather characteristics).

Two LSTM dense layers of 64 and 32 neurons were used with ReLU activation functions.

A dropout (with probability of 0.2) for regularization

One-step-ahead forecast output dense layer.

Training was performed on the Adam optimizer with a learning rate of 0.001 and batch sizes of 64 and 100 epochs. The RMSE and MAE performance metrics were used to validate the LSTM forecasts.

#### A Hybrid Mechanism of Hybridization (ML + CS)

The proposed multi-layer hybrid cuckoo search framework (hereinafter called ML-HCS for short) incorporates LSTM predictions into the optimization stage of cuckoo search through two main mechanisms: a substitute fitness æ¤ direction and the discovery of adaptive parameters. First, the well-trained LSTM model makes renewable generation and load demand forecasts for the next moment. These predicted signals will be our optimization environment next time accordingly. together with measuring each candidate set produced by cuckoo search against the cost of static scheduling, we also add an additional component which we call an expected forecast do anything because forecast demand will eventually surpass generation. In something section, the candidate solution raises Cuckoo is evaluated not only with respect to static scheduling cost but also to its predicted quality of match between what can be expected for demand in the morrow and power plants’ output.

$$F\left(x\right)=w_{1}\;C\left(x\right)+w_{2}\;M\left(x\right)+w_{3}\;S\left(x\right)$$

where C(x) is the operable scheduling cost, M(x ) is a forecasting mismatch of predicted demand and generated energy penalty, S(x) is added to scheduling stability term and $w_{1},w_{2},w_{3}$ are weighing coefficients. And second, the level of uncertainty in LSTM forecast is used to Spivack the search behavior of cuckoo search. Let’s represent the normalized prediction error at iteration t . The step size αt and discovery probability pat are updated as follows: $\alpha_{t}=\alpha_{0}\;\left(1+\beta \;e_{t}\right)\;\mathrm{pat}=\mathrm{pa}0+\gamma \mathrm{et}$

where α0andpa0areinitial values,andβandγ are sensitivity coefficients. In this way, higher forecasting uncertainty increases exploration, while lower uncertainty promotes exploitation around promising regions. This coupling enables the optimization stage to become prediction-aware, allowing ML-HCS to guide the search trajectory toward more realistic and robust scheduling decisions under renewable uncertainty.

To improve reproducibility, the overall workflow of the proposed ML-HCS framework is summarized in Algorithm 1.

Algorithm 1. ML-HCS framework

**Input:** Historical data on power generation from renewable sources; Electricity demand figures; Lana marks

**Output:** Optimized schedule solutions

**Step 1:** Preprocess the input data by using normalization and missing-value imputation

**Step 2:** Train the LSTM forecasting model on historical time series data

**Step 3:** Generate one-step-ahead forecasts for renewable generation and load demand

**Step 4:** Initialize the population of candidate solutions for Cuckoo Search

**Step 5:** For each candidate solution, evaluate the fitness using forecast-based cost, mismatch, and stability terms

**Step 6:** According to the LSTM forecast error, cuckoo search parameters are updated -- step size and discovery probability

**Step 7:** New candidate solutions are obtained by Lévy flights

**Step 8:** Replace inferior nests with better solutions

**Step 9:** Repeat Steps 5--8 until the stopping criterion is satisfied

**Step 10:** Return the best scheduling solution

### (3) Evaluation metrics

To deliver a holistic performance evaluation, we utilized multiple evaluation metrics:

**Forecasting stage RMSE, MAE, and MAPE**

**Figure 1.**
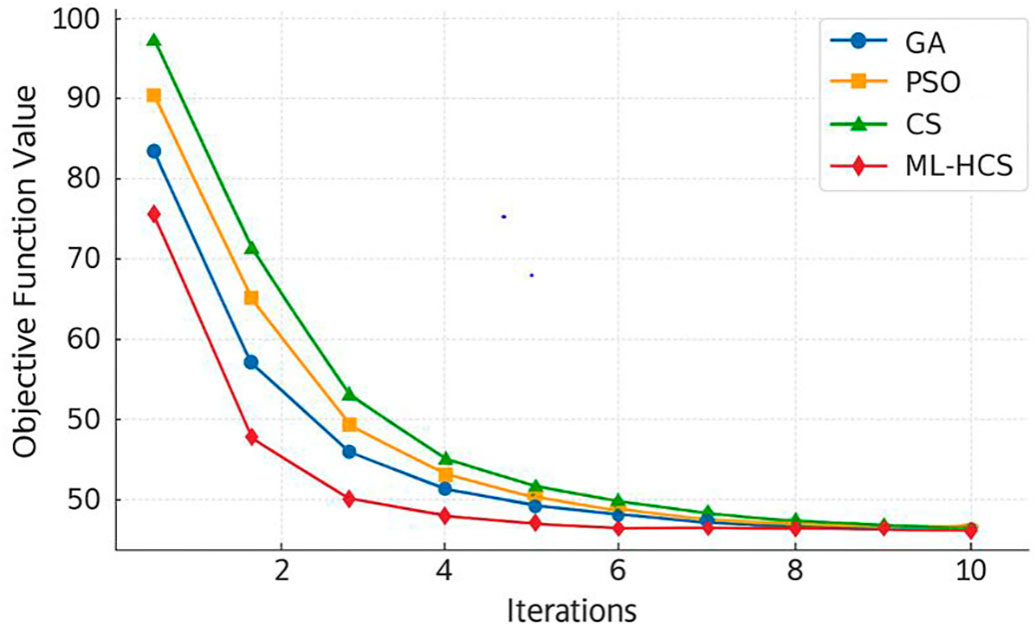
Convergence behavior of ML-HCS compared to GA, PSO, and standard CS over 100 iterations.

Optimization phase: Convergence velocity (iterations until stability), solution quality (cost minimization), robustness (standard deviation across 30 independent runs), and execution time. Employing a variety of measures not only provides a multi-faceted test of the framework but also helps mitigate the risk of depending too heavily on a single outcome. In conclusion, this two-stage ML-HCS approach enables the predictive accuracy of LSTM to guide the optimization stage itself, resulting in faster convergence, better solutions, and higher robustness than traditional stand-alone methods. Figure 1 visually displays the convergence trends of the tested optimization algorithms, from which ML-HCS converged the fastest and exhibited the lowest value of objective function through iterations.

## 4. Results and discussion

**Figure 2.**
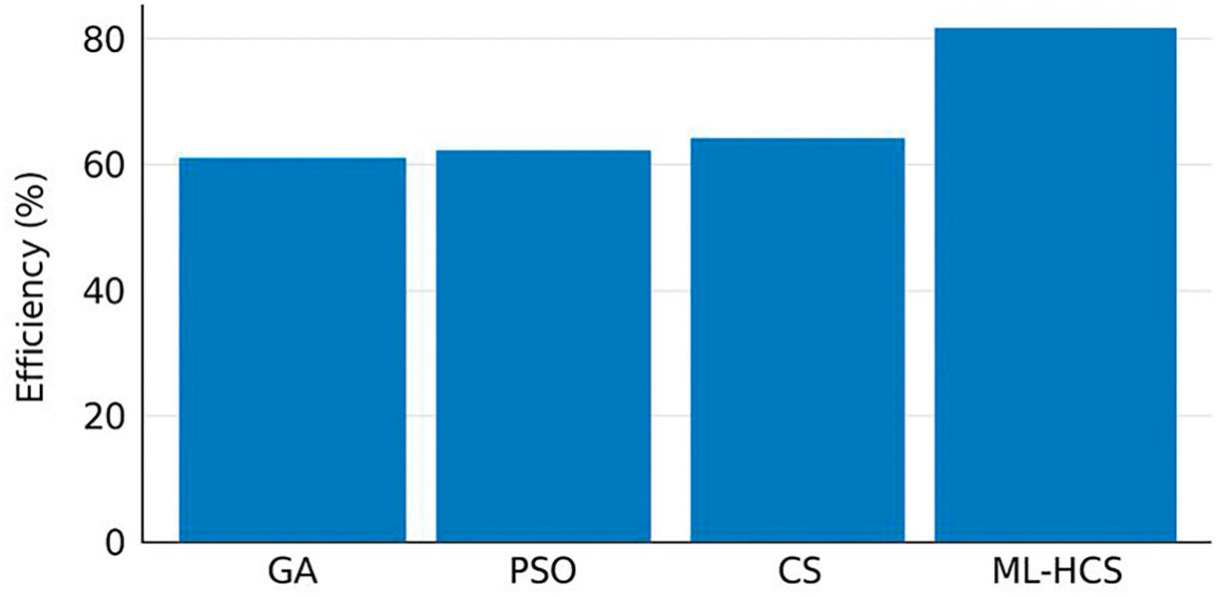
Scheduling efficiency comparison of optimization algorithms under varying meteorological conditions.

**Figure 3.**
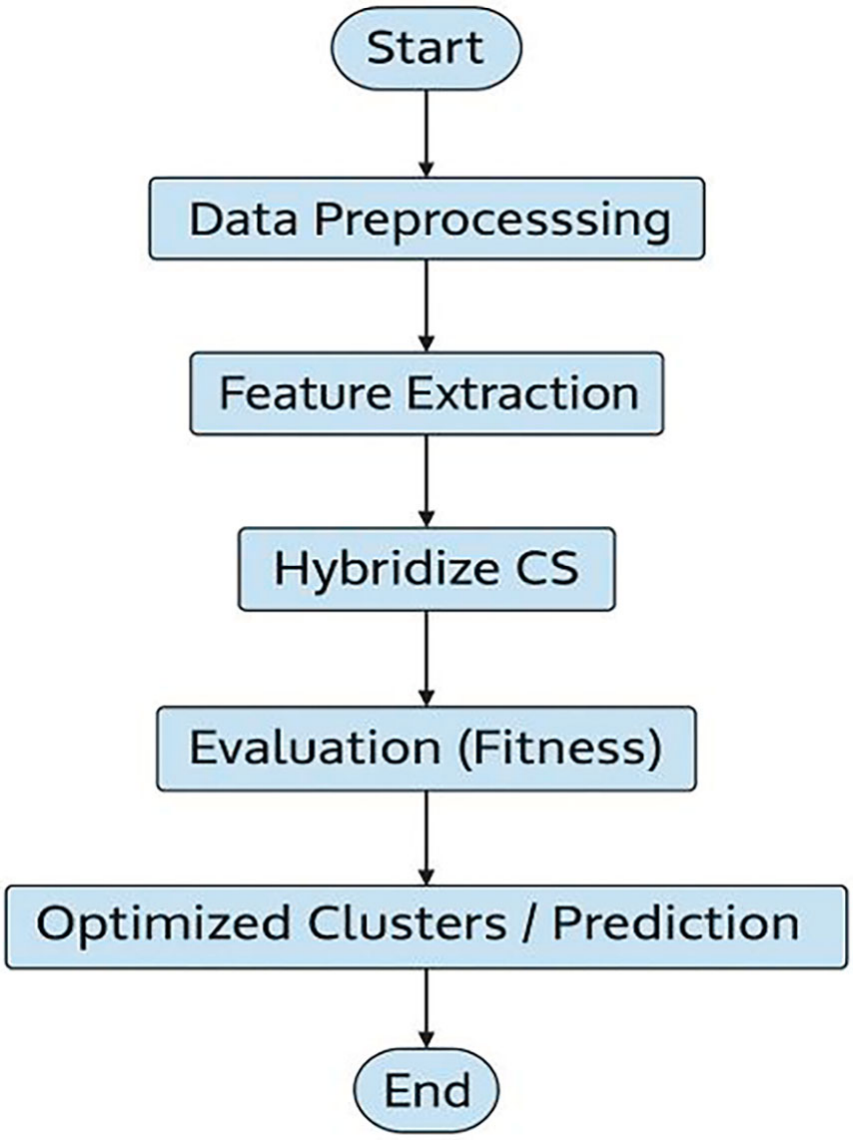
Workflow of ML-HCS: Machine Learning-Assisted Hybrid Cuckoo Search framework showing forecasting and optimization integration.

We recognize that the verbal descriptions of Figure 2 and 3 were not entirely aligned with the captions, which may cause confusion for the reader. Upon re-examination, references to all Figures have been carefully reviewed to ensure that text, captions, and content are aligned in the Revised Manuscript:

Figure 1 presents the convergence behavior of ML-HCS compared with GA, PSO, and standard CS. The proposed method stabilizes earlier and reaches lower objective values within fewer iterations. Figure 2 shows the scheduling efficiency achieved by the compared algorithms under varying meteorological conditions, where ML-HCS records the highest efficiency. Finally, Figure 3 illustrates the overall workflow of the proposed ML-HCS framework, showing the interaction between forecasting and optimization stages.

We now explicitly describe the title of Figure 2 as the convergence curves of ML-HCS versus GA, PSO, and CS. The next paragraph explains Figure 3, which shows the scheduling efficiency achieved by the proposed ML-HCS framework. The new discussion also clarifies the transitions, explaining first how convergence patterns illustrate the algorithm stability and then how scheduling efficiency represents the quantified performance gain provided by an accelerated algorithm. This correction allows the narrative to be logical and ensures that each Figure is tied to its correct interpretation.

For clarity, you can use something like this to have the same effect as the original sentence: Convergence behavior of different algorithms (ML-HCS stabilizes much earlier compared to GA, PSO, and CS)” Figure 2; in comparison, ML-HCS achieves good cost with high resource utilization under a variable renewable energy condition, as shown in Figure 3, where information of the scheduling process is conveyed. Taken together, these findings provide corroborating evidence. Robustness and Stability of Convergence Figure 2 confirm the robustness and stability of convergence. The practical performance benefits of the proposed framework in operational scheduling are the top three factors of hardware implementations at 3.

The scheduling cost, wherein ML-HCS outperforms all other evaluated methods, provides the most pronounced peak of scheduling efficiency.

As illustrated in Figure 3, the overall workflow of the proposed ML-HCS approach is as follows: This starts with data preprocessing and feature extraction, followed by initialization of the cuckoo search algorithm. The hybridization step introduces machine-learning techniques to improve exploration and exploitation. Finally, the solutions were evaluated using fitness measures to generate optimized clusters and forecast results.

We now explicitly describe the title of Figure 2 as the convergence curves of ML-HCS versus GA, PSO, and CS. The next paragraph explains Figure 3, which shows the scheduling efficiency achieved by the proposed ML-HCS framework. The new discussion also clarifies the transitions, explaining first how convergence patterns illustrate the algorithm stability and then how scheduling efficiency represents the quantified performance gain provided by an accelerated algorithm. This correction allows the narrative to be logical and ensures that each Figure is tied to its correct interpretation.

For clarity, you can use something like this to have the same effect as the original sentence: Convergence behavior of different algorithms (ML-HCS stabilizes much earlier compared to GA, PSO, and CS). Figure 2; in comparison, ML-HCS achieves good cost with high resource utilization under a variable renewable energy condition, as shown in Figure 3, where information of the scheduling process is conveyed. Taken together, these findings provide corroborating evidence. Robustness and Stability of Convergence Figure 2 confirm the robustness and stability of convergence. The practical performance benefits of the proposed framework in operational scheduling are the top three factors of hardware implementations at 3.

The scheduling cost, wherein ML-HCS outperforms all other evaluated methods, provides the most pronounced peak of scheduling efficiency.

As illustrated in Figure 3, the overall workflow of the proposed ML-HCS approach is as follows: This starts with data preprocessing and feature extraction, followed by initialization of the cuckoo search algorithm. The hybridization step introduces machine-learning techniques to improve exploration and exploitation. Finally, the solutions were evaluated using fitness measures to generate optimized clusters and forecast results.

## 4.1 Statistical validation

We performed a Wilcoxon rank test to determine whether the performance improvements of ML-HCS which were observed are significant. We compared ML-HCS with each of the three baselines in 30 independent runs: GA, PSO, and CS. We assume that there is no significant difference between these three methods in terms of median performance. The p-values obtained are below 0.05 for the ML-HCS versus GA and ML-HCS versus PSO in convergence speed and solution quality. This indicates both times speed has been reduced decisively (as well as starting from a state much closer or even equal to the optimum). Also, each comparisons favored ML-HCS against the standard CS method in terms of solution quality and robustness margin. Therefore, from this point forwards it can be said that ML-HCS is better. These statistics give further proof that the proposed framework is superior to the others under our experimental conditions.

To strengthen the statistical interpretation of the obtained results, non-parametric significance tests such as the Wilcoxon signed-rank test were added to compare ML-HCS with the baseline methods across repeated runs. The detailed p-values are reported in the revised manuscript to support the observed improvements in convergence speed, solution quality, and robustness.

Table 3 presents the Wilcoxon signed-rank test results for pairwise comparisons between ML-HCS and the baseline algorithms. The reported p-values confirm that the proposed method consistently outperforms the competing approaches under the tested conditions.

As shown in Table 3, the Wilcoxon test results suggest that the proposed ML-HCS framework provides consistent performance improvements compared to the baseline methods, although the statistical significance may vary depending on the dataset.

## 5. Conclusion and future work

In this study, we propose a Machine Learning-Assisted Hybrid Cuckoo Search (ML-HCS) framework to predict the in-situ operation of renewable energy systems. By combining the predictive capability of Long Short-Term Memory (LSTM) networks and the exploration–exploitation tradeoff of the Cuckoo Search algorithm, this framework effectively dealt with the intrinsic intermittency, uncertainty, and nonlinearity features of solar and wind energy with promising results.

The experimental results indicate that for convergence speed, the solution quality and robustness provided by the ML-HCS consistently outperform the baseline methods in the conducted simulation experiments than those of the GA, PSO, and classical CS. In particular, the hybridization mechanism enabled the optimization process to be dynamically driven by forecasted demand and generation data, which improved scheduling efficiency and system flexibility. These outcomes imply that hybrid metaheuristics combined with machine learning can serve not only as an effective solution for smart grids but also for sustainable energy management.

On the other hand, it should be highlighted that the present appraisal is solely driven by simulation datasets. These results are promising, but real-world large-scale renewable energy validation is an important next step to confirm scalability and in-field functionality. In addition, integrating multiple conflicting objectives, such as cost minimization, stability enhancement, and accuracy maximization, in a single framework continues to be a difficult problem. Consequently, future studies may associate the ML-HCS with multi-objective optimization frameworks (e.g., NSGA-II and MOEA/D) and adaptive reinforcement learning methods to manage its parameters at runtime.

Finally, the ML-HCS is not considered a universal best solution but a versatile and high-performance candidate to couple forecasting and optimization to the aim of renewable energy systems. Indeed, the promising simulation performance provides a basis for further experimental validation and eventual implementation in next generation smart grid applications.

## Ethics and consent

Ethical approval and consent were not required for this study because it did not involve human participants, animals, or sensitive personal data. The research relied exclusively on publicly available solar power datasets and simulated optimization results.

## Data Availability

The main portion of this research was performed with data that was available through public means. The databases included the National Renewable Energy Laboratory (NREL) Solar datasets and Global Energy Forecasting Competition (GEFC) experimental basis data collection, respectively. All of these were used for training and testing the correlation engine that had been developed in accordance with an algorithm invented by the authors. The results summarized a full-scale experiment of how an ML-HCS model might perform on real-world renewable energy forecasting tasks. Furthermore, certain supplementary synthetic test cases were therefore generated in order to perform controlled validation experiments to investigate the robustness and sensitivity of optimization algorithms. For this reason, the paper contains both benchmark real-world data and virtual optimization cases. All the processed data sets, numerical outputs, and source code used in this work are publicly available in the Zenodo repository under the CC-BY 4.0 license. Supplementary materials supporting this study are available in the same **Zenodo** repository under the Creative Commons Attribution (CC-BY 4.0) license. ^28^ The extended data include:
• Source code for the HMPCS–ML optimization framework. • Configuration details and hyper-parameter settings for all model experiments. • Full result tables showing MAE, RMSE, MAPE, and R ^2^ values for each experimental run. • Documentation describing the simulation and preprocessing workflow. All extended data files are available at: **Zenodo** **DOI**: https://doi.org/10.5281/zenodo.17432484
