# Supplementary material for: Machine Learning Assisted Hybrid Cuckoo Search for Predictive Optimization in Renewable Energy Systems
Source: F1000Res. 2026 Apr 24;15:436. Originally published 2026 Mar 25. [Version 2] doi: 10.12688/f1000research.172760.2 (PMC13110386; doi:10.12688/f1000research.172760.2)
Supplement: Peer review history

# Reviewer report: Reviewer response for version 2

**DOI:** 10.5256/f1000research.198588.r478880

**Published:** 2026-04-25

**Authors:** Zahir Abdul Haddi Hassan

**Affiliations:** 1 University of Babylon, Al Hillah, Iraq

I have carefully reviewed the revised version of the manuscript and the authors’ responses. Based on the improvements made, my final decision is: **Approved without further changes**.

 I appreciate the effort invested by both the authors and the editorial team.

Is the work clearly and accurately presented and does it cite the current literature?

Yes

If applicable, is the statistical analysis and its interpretation appropriate?

Yes

Are all the source data underlying the results available to ensure full reproducibility?

Yes

Is the study design appropriate and is the work technically sound?

Yes

Are the conclusions drawn adequately supported by the results?

Yes

Are sufficient details of methods and analysis provided to allow replication by others?

Yes

Reviewer Expertise:

NA

I confirm that I have read this submission and believe that I have an appropriate level of expertise to confirm that it is of an acceptable scientific standard.

# Reviewer report: Reviewer response for version 1

**DOI:** 10.5256/f1000research.190509.r471020

**Published:** 2026-04-11

**Authors:** Hussam Abid Ali Mohammed

**Affiliations:** 1 College of Education for Pure Sciences Dept. of Mathematics, University of Kerbala, Karbala, Karbala Governorate, Iraq

This research proposes a novel Machine Learning-Assisted Hybrid Cuckoo Search (ML-HCS) framework for predictive optimization in renewable energy systems. The study addresses key challenges associated with the stochastic, nonlinear, and intermittent nature of renewable sources such as solar and wind energy. The proposed method integrates Long Short-Term Memory (LSTM) networks for accurate time-series forecasting with a hybridized Cuckoo Search (CS) metaheuristic to enhance optimization performance.

 The framework operates in two stages: first, LSTM models generate predictive signals for energy demand and generation; second, these predictions guide the search process of the optimization algorithm, improving the balance between exploration and exploitation. Simulation results demonstrate that ML-HCS outperforms traditional approaches such as Genetic Algorithms (GA) and Particle Swarm Optimization (PSO), achieving faster convergence, higher solution quality, and greater robustness.

 The study presents a meaningful contribution by effectively combining forecasting and optimization within a unified architecture. However, some methodological aspects—particularly the mathematical formulation and statistical validation—require further clarification to ensure reproducibility and strengthen the scientific rigor. Overall, the work offers a promising and scalable solution for smart grid management and renewable energy optimization.

Is the work clearly and accurately presented and does it cite the current literature?

Yes

If applicable, is the statistical analysis and its interpretation appropriate?

Partly

Are all the source data underlying the results available to ensure full reproducibility?

Partly

Is the study design appropriate and is the work technically sound?

Yes

Are the conclusions drawn adequately supported by the results?

Yes

Are sufficient details of methods and analysis provided to allow replication by others?

Yes

Reviewer Expertise:

Operation Research

I confirm that I have read this submission and believe that I have an appropriate level of expertise to confirm that it is of an acceptable scientific standard.

# Response

**Published:** 2026-04-12

**Authors:** iraq abbas

**Affiliations:** Mathematics, University of Baghdad Al-Jaderyia Campus College of Science, Baghdad, Baghdad Governorate, Iraq

Dear Reviewer,

 We sincerely thank you for your valuable and constructive feedback.

 In response to your comments, we have strengthened the statistical analysis by repeating all experiments 10 times and reporting the results as mean ± standard deviation. Additionally, the Wilcoxon signed-rank test has been applied to confirm the statistical significance of the improvements achieved by the proposed ML-HCS method.

 Furthermore, the data availability section has been revised to clearly distinguish between real-world datasets and synthetic data used for robustness evaluation, ensuring full transparency and reproducibility.

 We believe that these revisions adequately address your concerns and improve the overall quality and rigor of the manuscript.

 Sincerely,  

 The Authors

# Reviewer report: Reviewer response for version 1

**DOI:** 10.5256/f1000research.190509.r471019

**Published:** 2026-03-31

**Authors:** Zahir Abdul Haddi Hassan

**Affiliations:** 1 University of Babylon, Al Hillah, Iraq

1.Methodological Detail & Replication: While the integration of LSTM with Cuckoo Search is promising, the manuscript would benefit from providing more precise mathematical formulations or pseudo-code detailing how the LSTM predictive signals specifically guide the CS search trajectory and how the adaptive parameters are dynamically updated. This additional detail is essential to ensure full reproducibility of the hybrid framework by other researchers.

 2. Statistical Analysis: The evaluation relies primarily on a percentage-based comparison of means and standard deviations. To strengthen the statistical interpretation, it is recommended to include significance testing (e.g., p-values or Wilcoxon tests) to confirm that the observed improvements in convergence and solution quality are statistically significant Data Consistency: There appears to be a slight inconsistency regarding the nature of the source data; the methodology cites the use of real-world benchmark datasets such as NREL and GEFC , whereas the "Data Availability" section describes the supporting data as "synthetic and simulated". Clarifying this distinction would enhance the transparency and reliability of the reported results.

Is the work clearly and accurately presented and does it cite the current literature?

Yes

If applicable, is the statistical analysis and its interpretation appropriate?

Yes

Are all the source data underlying the results available to ensure full reproducibility?

Yes

Is the study design appropriate and is the work technically sound?

Yes

Are the conclusions drawn adequately supported by the results?

Yes

Are sufficient details of methods and analysis provided to allow replication by others?

Yes

Reviewer Expertise:

NA

I confirm that I have read this submission and believe that I have an appropriate level of expertise to confirm that it is of an acceptable scientific standard, however I have significant reservations, as outlined above.

# Response

**Published:** 2026-04-12

**Authors:** iraq abbas

**Affiliations:** Mathematics, University of Baghdad Al-Jaderyia Campus College of Science, Baghdad, Baghdad Governorate, Iraq

Dear Reviewer,

 Thank you for your valuable comments.

 We have enhanced the methodological section by clearly describing the integration between LSTM and Cuckoo Search, including mathematical formulation and adaptive parameter updates. Statistical validation using the Wilcoxon test has been added, and data consistency has been clarified by distinguishing between real-world and synthetic datasets.

 We believe that these revisions fully address your concerns.

 Sincerely,  

 The Authors
